# Does Acute Stress Impact Declarative and Procedural Learning?

**DOI:** 10.3389/fpsyg.2020.00342

**Published:** 2020-03-26

**Authors:** Ranin Ballan, Yafit Gabay

**Affiliations:** ^1^Department of Special Education, University of Haifa, Haifa, Israel; ^2^Edmond J. Safra Brain Research Center for the Study of Learning Disabilities, University of Haifa, Haifa, Israel

**Keywords:** category learning, delay feedback, feedback-based learning, hippocampus, incremental learning of stimulus-response associations, procedural learning, stress, striatum

## Abstract

It is well established that acute stress can influence memory function, yet its influence may differ across memory systems. Whereas stress sometimes exerts a negative influence on declarative learning, it does not necessarily harm learning in general, as demonstrated in the case of procedural learning. Probabilistic category learning is mediated by the striatum, but delaying feedback by a few seconds shifts learning to become more hippocampal-dependent. Here, we examined the influence of acute stress on this type of learning, under different conditions that favor either procedural-based (immediate feedback) vs. declarative-based (delayed feedback) learning. Sixty-two participants randomly assigned to either stress or non-stress groups, performed a probabilistic category learning task, in which they were instructed to learn associations between cues and outcomes under different feedback conditions (immediate feedback, short-delayed feedback, and long-delayed feedback). Acute stress was induced by the Maastricht Acute Stress Test (MAST), and stress levels were gauged by Galvanic Skin Response (GSR) measures and a self-reported questionnaire. Results showed that although the MAST was effective in inducing stress, this did not harm learning in either of the feedback conditions. These findings suggest that not all hippocampal-based learning types are negatively influenced by stress.

## Introduction

It is common to distinguish between several types of learning and memory systems (Squire, 1992). The declarative based memory system (“knowing that”) refers to the acquisition of semantic and/or episodic memory and depends on the integrity of medial temporal lobe structures, including the hippocampus. The procedural memory system (“knowing how”), on the other hand, subserves the acquisition of skills and procedures and is supported by the basal ganglia, and particularly the striatum. A burgeoning literature suggests that stress exerts different influences on distinct learning and memory systems. In particular, stress has been shown to shift the learning process to rely less on medial temporal structures and more on the striatal (Schwabe and Wolf, 2013; Goldfarb and Phelps, 2017).

Evidence for stress-induced trade-offs between memory systems was initially shown in navigation tasks, where stress induction yielded shifts from hippocampal-based learning strategies to striatal-based learning strategies (Schwabe, 2013). Yet, the influence of stress on multiple memory systems goes beyond navigation tasks. Schwabe and Wolf (2012) examined the influence of stress on the weather prediction task, a classic procedural learning task that has typically been shown to engage the striatum. Stress was triggered by the Socially Evaluated Cold-Pressor Test (SCEPT; Schwabe et al., 2008b), in which participants are required to immerse their hand in ice water while being informed that their facial expressions will be filmed during hand immersion. Although learning performance was not influenced by stress, Schwabe and Wolf (2012) found that stress changed participants’ learning strategy from a single-cue-based declarative strategy to a multi-cue-based procedural strategy. Furthermore, the neuroimaging data in the same study revealed hippocampal activity during task performance devoid of the stress manipulation, whereas striatal activation was observed when performing the task under stressful conditions. Stress-induced effects on further procedural striatal-based learning tasks were documented in visual category learning (Markman et al., 2006; Ell et al., 2011; McCoy et al., 2014), speech category learning (Maddox et al., 2016), and reinforcement learning (Cavanagh et al., 2010; Petzold et al., 2010). In the study conducted by Markman et al. (2006), participants were convinced that their performance would determine whether they and a fabricated companion would earn a monetary bonus. Such a type of performance pressure improved the learning of multi-dimensional categories whose learning is based on the procedural learning system, but impaired the learning of rule-based categories whose learning is assumed to depend on the declarative learning system. A similar finding was also observed in procedural learning of speech categories (Maddox et al., 2016). Stress was also found to influence procedural reinforcement learning (Petzold et al., 2010). In the study of Petzold et al. (2010), although psychosocial stress (i.e., the Trier Social Stress Test; TSST; Kirschbaum et al., 1993) did not influence task acquisition, it led participants to use negative feedback significantly less during learning while it failed to affect the use of positive feedback. Based on their findings, the authors concluded that stress leads to an increase of dopamine levels in the brain, thus reducing the reinforcement signal of negative feedback. In addition, a study that examined the effect of stress (cold pressor test) on computational reinforcement learning strategies found that stress attenuated the use of goal-directed (model-based) strategies but not of habitual strategies (model free) during decision making (Otto et al., 2013). Finally, stress was found to enhance motor procedural learning. In the study conducted by King (2013), better performance on a sequential learning task was observed among participants who were exposed to stress compared to participants in the non-stress condition. Consistent with these findings, stress was found to prompt dependence on habitual behaviors at the cost of flexible, goal-directed behaviors (Schwabe et al., 2007; Schwabe and Wolf, 2009). These studies suggest that stress may have no influence on, or in some cases may even benefit striatal-based learning.

In contrast, stress negatively influences hippocampal-based learning (Kim et al., 2015). In the study conducted by Newcomer et al. (1994), stress induced by a pharmacological treatment (dexamethasone) was shown to impair delayed recall (at day 4) in a verbal declarative memory task (memorizing a paragraph). Others have demonstrated that psychosocial stress (TSST) impaired participants’ ability to distinguish words presented for study from non-presented lure words that were semantically related, therefore increasing the likelihood of forming false memories (i.e., an ability that is attributed to hippocampal and prefrontal function; Payne et al., 2002). Similarly, Kuhlmann et al. (2005) showed that participants who were exposed to the same psychosocial stressor performed significantly worse when required to memorize word lists learned 24 h earlier, suggesting that stress impairs memory retrieval. Other studies found no significant differences in performance between stress and non-stress conditions but rather associations between poorer performance on declarative memory tasks and an elevated stress-induced cortisol increase (by the TSST) within a group that was exposed to stress (Kirschbaum et al., 1996; Wolf et al., 2001). Stress (induced either by the TSST or by SCEPT) was also found to impair performance on working memory tasks measured by the backward digit-span test (Kuhlmann et al., 2005) and context-dependent memory (Schwabe et al., 2009). Consistent with these findings, individuals diagnosed with post-traumatic stress disorder are impaired in tasks that require verbal recall (Bremner et al., 2000).

Notably, however, not all studies are consistent with the notion that stress impairs hippocampal-based learning. Several studies indicate that stress can actually improve hippocampal-based learning. For example, rats subjected to retrained stress displayed enhanced hippocampus-dependent contextual fear conditioning (Cordero et al., 2003). In humans, psychosocial stress (TSST) improved participants’ performance on an explicit spatial memory task in which subjects were required to learn a route on a map (Luethi et al., 2009). Similarly, Duncko et al. (2007) observed that men exposed to a physiological stressor (cold pressor test; CPT) performed better on a spatial learning task. Prior exposure to a psychosocial stressor (TSST) can also facilitate memory of emotional words and this is observed particularly for negative rather than positive words (Schwabe et al., 2008a). In addition, introducing a temporal gap (25 or 90 min) between stress induction (given 24 h after learning a list of words) and test impairs memory retrieval, but performance is unaffected when memory retrieval is tested immediately after the stress induction (Schwabe and Wolf, 2014). Additional studies reported no performance differences between stress and non-stress groups but showed that participants who were high responders to a psychosocial stress manipulation (TSST) performed better on declarative memory tasks such as word recall or the Rey Auditory Verbal Learning Test (Domes et al., 2002; Nater et al., 2007). Thus, whether all types of hippocampal-based learning are negatively influenced by stress is still debated.

There are several explanations for the discrepancies across studies, such as the level of stress (acute vs. chronic), different cognitive processes involved in a given task (for example, working memory demands might differ across tasks), the time point in which stress is delivered (acquisition, consolidation, or retention phase), as well as the interval between stress induction and the learning task (for reviews see, Joëls et al., 2006; Schwabe et al., 2012; Sandi, 2013; Cadle and Zoladz, 2015; Quaedflieg and Schwabe, 2018). The two latter factors seem to be particular important in light of the temporal dynamics model of stress (Diamond et al., 2007). According to this model, when stress occurs in close temporal proximity to learning, long-term memory retrieval will be enhanced. When the stressor is temporally separated from the learning or occurs prior to retrieval, long-term memory will be impaired. The assumption is based on the observation that neurotransmitters and hormones released during stress exert rapid and slower effects. Stress leads to induced neurochemical activity that results in enhancement of hippocampal function and learning. However, as time and/or stressor carry on neurochemical activity leads to an inhibition of hippocampal function and learning during this period is presumed to be impaired. Therefore, stress has contrary effects on learning processes, depending on the timing of the events. Consistent with this model, exposure to stress given shortly before a retrieval test impairs retention but has an enhancing effect on memory encoding or consolidation (Cadle and Zoladz, 2015). In addition, a longer interval between stress induction and learning results in impaired performance compared to a situation in which stress is triggered immediately before learning (Smeets et al., 2009). This could explain the discrepancies between the studies reported above with regard to hippocampal-based learning.

Although the studies reviewed above suggest that stress can sometimes promote striatal-based learning while impairing hippocampal-based learning, complex skills are likely to involve a mixture of procedural and declarative processes that interact in complex ways (Jiménez and Méndez, 1999; Sun et al., 2005). Even well-established procedural learning tasks are likely to involve both processes (Sun et al., 2001). Examination of the interaction between these two processes can be achieved, for example, either by varying task instructions, by employing a secondary task or by manipulating the statistical structure of the task (Sun et al., 2005). Explicit instructions are likely to encourage the involvement of declarative-based memory systems, whereas the use of a dual task encourages reliance on procedural-based memory systems.

It has recently been shown that feedback timing can also modulate the engagement of neural systems and, as a result, influence behavioral outcomes. Specifically, feedback based learning is typically sensitive to striatal function, however, delaying the feedback between stimuli and responses modifies the learning process to become more hippocampal-dependent (Foerde and Shohamy, 2011). The assumption is that phasic dopamine responses to feedback are observed approximately 100 ms following a reward (Redgrave and Gurney, 2006). These responses are thought to facilitate learning by enabling cortico-striatal plasticity, presumably by supporting reward-related associations with relevant responses or stimuli (Reynolds and Wickens, 2002). Consistent with this assumption, animal data shows that when rewards are given after a long delay this results in reduced dopaminergic activity compared with rewards of the same value given after a short delay (Roesch et al., 2007). In humans, categorization afforded solely based on procedural learning is impaired under delayed feedback conditions but not when categorization is based on declarative learning (Maddox et al., 2003; Maddox and David, 2005; Chandrasekaran et al., 2014). In addition, other procedural learning tasks such as the learning of artificial grammar learning rules are impaired under delayed feedback conditions compared to immediate ones (Opitz et al., 2011). Using a neuroimaging method (fMRI), Foerde and Shohamy (2011) observed striatal activity during a probabilistic learning task in which feedback for choice outcomes was immediate, whereas a hippocampal response was observed when feedback was delayed by a few seconds. This trade-off between memory systems based on feedback timing is also supported by studies with patients. In disorders characterized by altered dopaminergic function in the striatum, learning is impaired when feedback is immediate but is intact when it is delayed by a few seconds (Foerde et al., 2012; Gabay et al., 2018), whereas the opposite pattern is observed among people with hippocampal damage (Foerde et al., 2013b). Taken together, these findings suggest that feedback timing is an important factor when considering the relative engagement of memory systems.

In the present study, our goal was to examine the influence of stress on different memory systems at the behavioral level. However, as mentioned earlier, this goal is challenging, because the output of almost every behavior likely includes output from all memory systems. Our approach for addressing this challenge was to use a probabilistic learning task in which feedback timing was manipulated. This approach is motivated by a literature showing that performance in the task we used can be modulated by feedback timing (Foerde and Shohamy, 2011; Foerde et al., 2013a, b; Gabay et al., 2018). In particular, a double dissociation was found in patient studies that used the same task. Patients with basal ganglia dysfunction were impaired in the immediate feedback condition but not in the delayed one (Foerde et al., 2013a) whereas the opposite pattern was observed in patients with hippocampal damage (Foerde et al., 2013b). Furthermore, previous research has demonstrated that the task used here is associated with hippocampal and striatal activations under delayed and immediate feedback conditions, respectively (Foerde and Shohamy, 2011). These studies support the notion that feedback timing plays a critical role in modulating the involvement of memory systems. However, the present study did not include a neural measurement that can indicate the involvement of the different neural systems or behavioral assays through which the recruitment of different memory systems could be identified. Hence, it is difficult to demonstrate the involvement of the different memory systems in the current investigation. Although previous studies that employed the current manipulation did demonstrate its ability to bias behavior toward procedural or declarative memory systems, if for any reason this was not the case in the current study, our investigation also has implications regarding the effects of stress in real-world learning environments in which feedback is not always delivered immediately.

Participants in a stress-induced group and in a control group performed a probabilistic category learning task under different feedback conditions (immediate/delayed). Delaying feedback is likely to shift striatal-based learning to become more hippocampal-dependent. Therefore, we hypothesized that if stress impairs hippocampal-based learning, the non-stressed group will outperform the stressed group under the delayed feedback conditions. In addition, it has been suggested that acute stress leads to an increase in dopamine levels in the brain, thus reducing the reinforcement signal of negative feedback (Petzold et al., 2010). Low levels of striatal dopamine (Parkinson’s patients off medication) are related to better ability to learn from negative (Frank et al., 2004) or delayed feedback (Foerde et al., 2012; Gabay et al., 2018). Therefore, one could hypothesize that stress would lead to better learning under immediate feedback conditions.

## Materials and Methods

### Participants

Seventy-three participants (age range 18–35) were recruited at the University of Haifa. Prior to the training session, they were required to provide background information about their age, gender, educational level, and handedness. They received payment (50 new Israeli shekels, the equivalent of approximately $14 US), had normal or corrected-to-normal vision, and reported normal hearing and having no learning disability. Participants were randomly assigned to one of two experimental groups: stress (*N* = 41, 25F, 16M) or non-stress (*N* = 32, 23F, 13M), and most were right handed (5 and 4 left handed in the stress and non-stress groups, respectively). The study was approved by the Ethics Committee of the Faculty of Education at the University of Haifa and adhered to the Helsinki Declaration. All participants signed an informed consent form and were debriefed regarding the manipulation following the experimental session.

### Stress Manipulation

The Maastricht Acute Stress Test (MAST) served for stress manipulation, similar to the procedures carried out in Smeets et al. (2012). It consisted of a 5-min preparation phase and 10 min in which acute stress, which encompassed physical, social, and arithmetic components, was delivered. During the preparation phase, participants were seated in front of a computer screen and were instructed about the upcoming task using a PowerPoint presentation. In the second phase, participants were required to immerse their dominant hand (including the wrist) in ice-cold water (0–3 C°) at different time points that were randomly chosen by the computer but did not exceed 90 s. In between hand dipping instances, participants were instructed to place their arm on a towel alongside the water bowl and to perform a mental arithmetic test, which consisted of backward counting beginning from 2043 in steps of 17, as quickly and as accurately as possible. Negative feedback was given to participants every time they made a mistake and in such cases they were required to begin the computations over again from 2043. Participants were required to perform the mental computations until the computer signaled the beginning of the next hand dipping trial, which would take at least 45 s. Participants were also told that during the hand dipping task, their facial expressions would be recorded for the purpose of evaluating facial expressions of pain at a later stage, and they provided written consent for this (in fact, facial expressions were not recorded). Participants in the non-stressed group were required to immerse their hands in lukewarm water (37–35 C) and were not required to perform any mental arithmetic test.

### Stress Manipulation Assessment

Stress levels were assessed using a self-report questionnaire and galvanic skin response (GSR) measures.

#### Self-Report Questionnaire

The self-report questionnaire contained eight items (e.g., to what extent do you feel alert right now?), and each item was rated on a 5-point Likert scale ranging from 1 = not at all to 5 = very much) (please see Supplementary Material). Each participant was assigned a self-report stress score by summing the values assigned to each item, yielding a score in the range of 8 to 40, with higher scores indicating more severe stress-related symptoms. Each participant was required to complete the self-report questionnaire three times during the experimental session: before the stress manipulation, after the stress manipulation, and at the end of the probabilistic learning task.

#### Galvanic Skin Response

**FIGURE 1:**
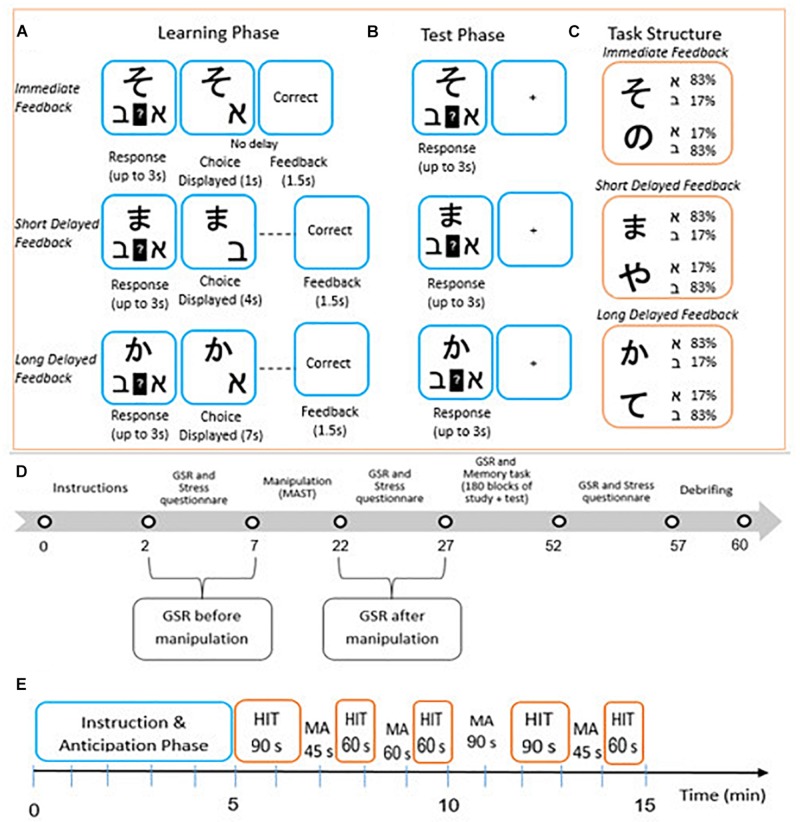
Probabilistic category leaning task adapted from Gabay et al. (2018), modeled after Foerde and Shohamy (2011). Participants used trial-by-trial feedback to learn which of two Hebrew letters (ℵ or ℶ) is associated with one of six different Asian characters (Learning phase, **(A)**. For one set of Asian characters, feedback was presented immediately (0 s) after choice display. For another set of Asian characters, feedback was presented with a short (3 s) or long delay (6 s) after choice display. After the learning phase was terminated, participants completed a test phase in which they continued to make predictions about associations between letters and characters (Test phase, **B**). However, corrective feedback was no longer provided and the timing of all trial events was equal across trial types. Each Asian character was associated with one Hebrew letter on 83% of trials and with the other Hebrew letter on 17% of trials **(C)**. Different time points across the experiment in which stress questionnaire and GSR were measured **(D)**. Different time points across the hand immersion task in which participants in the stress group were either required to immerse their hand in cold water or perform mental calculations **(E)**.

Stress levels were also measured via GSR recordings by applying sensors to the non-dominant hand of the participant. One sensor was attached to the middle finger and the other sensor to the ring finger of the left hand. As GSR provides indices of skin conductivity mediated by the autonomic nervous system, the information obtained can serve as an indication of psychological or physiological arousal (Guez et al., 2016). GSR recording was performed using BioNex 8SLT Chassis Assembly and a wireless recording device (manufactured by Mindware). Skin conductance was recorded at a rate of 500 samples/second using a two finger Touch proof Snap Lead- Green (Model 93-0404-00)/GSR sensor and two electrode sets of 100 Disposable GSC Electrodes (Model 93-0102-00). The conductance measurement was performed at different time-points during the experiment: (1) before the stress manipulation, (2) immediately after the stress manipulation, (3) during performance of the probabilistic learning task, and (4) at the end of the probabilistic learning task. GSR measurements before/after stress manipulation and at the end of the task lasted 3 min. The GSR measurement during the task lasted 20 min. Raw data was used for averaging. For each subject, a mean score of SC was calculated four times: before and after the manipulation, during the task, and at the end of the task (see Figure 1D for schematic representation of the procedure) similar to the study of Guez et al., 2016.

### Probabilistic Learning Task

Participants carried out a probabilistic category learning task, based on the research of Gabay et al. (2018) modeled after Foerde and Shohamy (2011). During the learning phase, in each trial participants were presented with one of six Asian characters and were asked to predict with which of the Hebrew letters the Asian character is associated (see Figure 1A). The probabilities used were such that each Asian character predicted that one of the two Hebrew letters would yield a rewarding outcome on 83% of trials and the other on 17% of trials (see Figure 1C). After participants made a response, feedback was presented after a fixed delay of either 0 s (immediate feedback), 3s (short delayed feedback), or 6 s (long delayed feedback). The task was built in such a way that each Asian character was associated with one of the delay intervals (two characters allocated at random for each delay). Trial types for each feedback condition were interleaved throughout the training. Participants were required to provide a response within a time frame of 3 s. After participants’ response, the chosen outcome appeared on the screen for 1 s (followed by a delay period of 0, 3, or 6 s) alongside the cue to minimize working memory demands. Therefore, the critical manipulation was the time interval between participants’ responses and feedback. Because there was a possibility that response times could vary across trials and participants, the overall trial length (character onset to feedback end) could vary, but the time between responses and feedback remained fixed within each trial type. After the delay period, feedback in the form of the words “correct” or “incorrect” was presented on the screen for 1.5 s. The behavioral measurement of performance in the task consisted of the percentage of successful choices for each feedback delay condition (i.e., selecting the letters that lead to correct feedback for each cue). In the learning phase, participants completed 180 trials of the task, followed by a test phase that was similar to the training phase except that no feedback was provided to participants after responding and it began immediately following the learning phase, upon which instructions were presented on the screen (see Figure 1B).

### Procedure

The experiment was conducted in a quiet room. Stimulus presentation and recording of response time and accuracy were controlled by an E-Prime computer program (Schneider et al., 2002). Participants were first assigned to a stress or non-stress condition and, following the stress manipulation, they completed the stimulus-response learning task (see Figure 1). The experiment was conducted in a single session that did not exceed 1 h.

Figure 1D presents a summary of the session timeline. After arriving at the laboratory, written informed consent was obtained from the participants, stating that they would be required to place their hand in a bowl of water during parts of the experiment. Participants in both groups were then required to complete a background questionnaire that included questions about gender, age, academic background, and dominant hand. Measures of stress levels were obtained via the abovementioned self-report questionnaire and skin conductance during the following time points throughout the experiment: before and after the stress manipulation, during the stimulus-response learning task, and at the end of the learning task (the self-report questionnaire was not administered during the stimulus-response learning task). After employing the MAST task and for the second time, participants were asked to complete a self-report questionnaire to obtain baseline ratings of their current mood, including measurement of conductance for 3 min. Subsequently, during the course of the probabilistic category learning task, an additional measurement of conductance was carried out. Upon termination of the stimulus-response learning task, participants were required to once again complete the self-report questionnaire and undergo skin conductance recording. At the end of the experiment, all participants were debriefed and remunerated for their participation. The entire session lasted approximately 50 min.

Our main objective was to assess whether and how stress induction influences probabilistic learning. We first examined whether the groups in fact differed in stress-related responses, as measured by (1) the self-report questionnaire and (2) GSR recordings, using a non-parametric test and *t* tests. Second, we examined possible differences between the stress and non-stress groups in the stimulus-response learning task as a function of feedback timing in both learning and test phases, using mixed-effects ANOVAs. For the stimulus-response learning task, we also calculated a Bayes factor (BF) for each effect of interest. The Bayes factor states the ratio between the evidence supporting the hypothesis relative to the null hypothesis (Dienes, 2008), such that a Bayes factor with a value of less than 1/3 indicates support for the null hypothesis. In contrast, a Bayes factor over 3 suggests that the analysis supports H1. Bayes factors were calculated using JASP – a free software for statistical analysis.^1^

## Results

Seven participants within the stress group were omitted from the analysis either due to technical problems (*N* = 2) or since they asked to stop the hand immersion task (*N* = 5). In addition, participants in the stress group who were the least responsive to stress, based on the objective and subjective measures, were excluded from the analyses. We identified these individuals based on changes in self-reported questionnaire scores and GSR measurements from before the stress manipulation to after the stress manipulation (difference scores).

In particular, we first standardized the GSR scores (Z scores) from the time before the stress manipulation to the time after the stress manipulation. Next, using these values, participants were divided into three levels: high responders, medium responders, and low responders, such that there were at least 1/3 participants in each level. We repeated this analysis using the self-questionnaire score. Participants placed at the third lowest range in both difference scores were not included in the analysis (*N* = 4). Therefore, the final sample included 30 participants in the stress group and 32 participants in the non-stress group.

### Subjective Response to Stress

**FIGURE 2:**
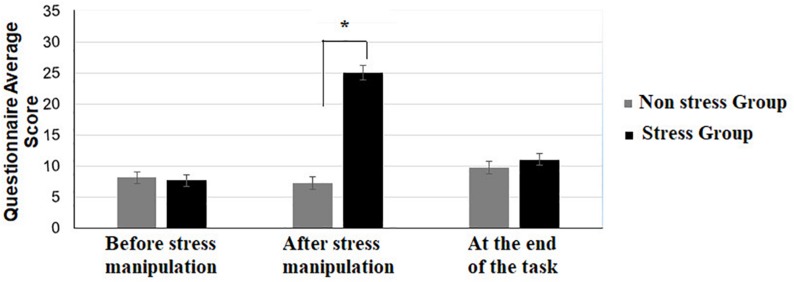
Average score on the self-report questionnaire of the stress and non-stress groups, before/after the stress manipulation and at the end of the stimulus-response learning task. Asterisks represent the following: *p 0.05 and error bars represent standard errors of the mean.

Participants’ subjective responses throughout the session were explored by comparing the two groups at each time point using a non-parametric Mann-Whitney U test (see Figure 2). A significant difference between the two groups was evident only after the stress manipulation: [*Z* = 0.43, *p* = 0.66] before the stress manipulation, [*Z* = −6.69, *p* < 0.001] (with Bonferroni correction) after the stress manipulation, [*Z* = −1.03, *p* = 0.30] at the end of the task (Figure 2).

### Galvanic Skin Response (GSR)

**FIGURE 3:**
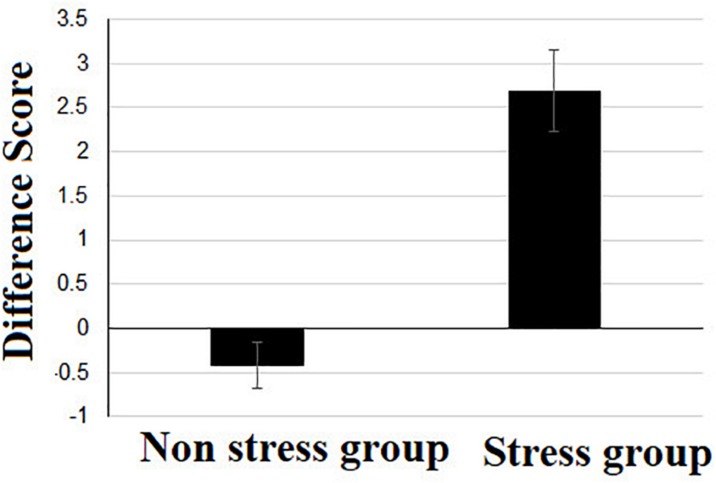
Difference score of skin conductivity measure, calculated by subtracting the pre-stress skin conductivity value from the post-stress skin conductivity value.

In order to examine the influence of stress on GSR levels we calculated a difference score for each individual (post stress minus pre stress) and then compared these values between stress and control participants using a *t*-test for independent groups (see Figure 3). There was a significant difference between the two groups *t* (1, 61) = 6.01, *p* = 0.00, such that the difference score for GSR levels in the stress group (*M* = 2.69, *S.D*. = 2.51) was significantly higher from that of the non-stress group (*M* = −0.42, *S.D*. = 1.49).

### Probabilistic Category Learning Task

#### Learning Phase

**FIGURE 4:**
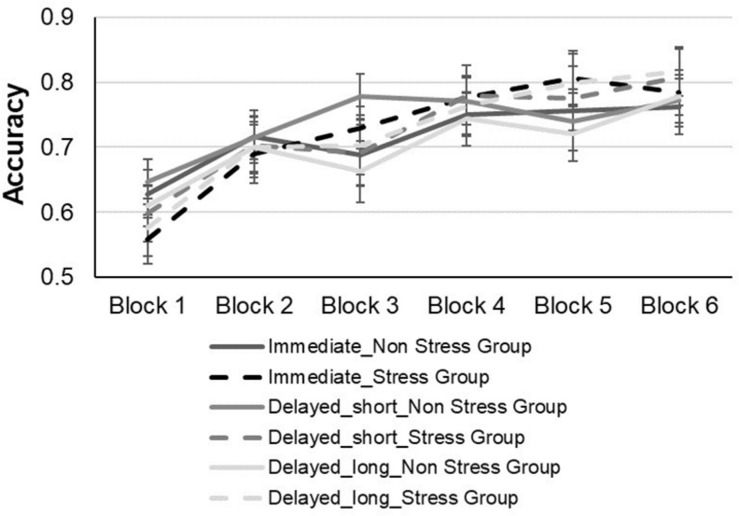
Accuracy performance of the stress and non-stress groups during the test phase across all feedback conditions.

For the learning phase, an analysis of variance (ANOVA) was conducted, with block (1–6) and feedback type (immediate, short-delayed, and long-delayed feedback conditions) as within subject factors, and group (stress vs. non-stress) as a between subject factor using mean proportion of correct responses during the learning phase as the dependent variable (see Figure 4).^2^ The main effect of group was not significant [*F*(1,60) = 0.5, *p* = 0.83, η*p^2^* = 0.00007, BF = 0.190], such that accuracy during the learning phase did not differ significantly between the two groups. There was a significant main effect of block [*F*(3,500) = 29.33, *p* < *0.001*,η*p^2^* = 0.32, BF = 6.390 + 23], such that accuracy rates increased as training progressed, yielding a significant linear trend [*F*(1,60) = 60.42, *p* < *0.001*]. Thus, as the blocks progressed, there was a linear increase relative to the percentage of accuracy in performing the task, indicating that learning had occurred. The main effect of feedback was not significant [*F*(2,120) = 0.29, *p* = 0.74; η*p^2^* = 0.004, BF = 0.02]. In addition, the interaction of block by group was not significant, [*F*(5,300) = *0.91, p* = 0.47 η*p^2^* = 0.01, BF = 0.009], as well as the interaction of block by feedback type, [*F*(10,600) = 97, *p* = 0.46, η*p^2^* = 0.01, BF = 0.001] and the interaction of feedback type by group, [*F*(2, 120) = 37, *p* = 0.68 η*p^2^* = 0.006 BF = 0.05]. Also, the triple interaction between block × feedback type × group was not significant [*F*(10, 600) = *0.60*, *p* = *0.81*,η*p^2^* = 0.009, BF = 0.003].

#### Test Phase

**FIGURE 5:**
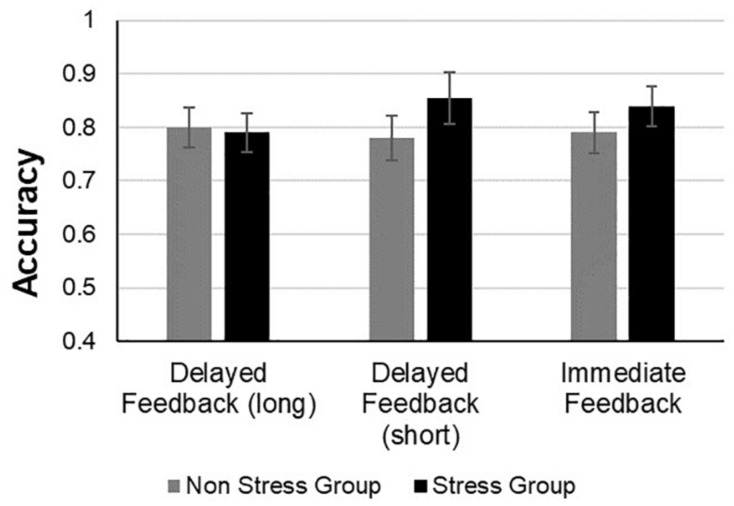
Accuracy performance of the stress and non-stress groups during the learning phase across all feedback conditions.

For the test phase, an analysis of variance (ANOVA) was conducted with feedback type (immediate feedback, short delayed feedback, and long delayed feedback) as the within subject factor, and group (stress vs. non-stress) as the between subject factor and mean proportion of correct responses during the test phase as the dependent variable (see Figure 5). The main effect of group was not significant [*F*(1,60) = 0.67, *p* = 0.41, η*p^2^* = 0.01, BF = 0.128], implying that the stress group did not differ significantly from the non-stress group with regard to accuracy in the test phase. Furthermore, neither the main effect of feedback type [*F*(2,120) = 1.003, *p* = 0.37, η*p^2^* = 0.01, BF = 0.367] nor the interaction between group and feedback type [*F*(2,120) = 0.32, *p* = 0.72, η*p^2^* = 0.005, BF = 0.126] were significant.

## Discussion

Burgeoning research indicates a trade-off between hippocampus vs. striatal based learning and memory systems (Schwabe and Wolf, 2013; Goldfarb and Phelps, 2017). Delaying feedback by a few seconds can shift procedural learning to become more hippocampal-based (Foerde and Shohamy, 2011; Arbel et al., 2017; Lighthall et al., 2018). Using such a feedback-timing manipulation, we aimed to determine how stress influences other types of hippocampal-based learning beyond its influences on semantic, episodic, and spatial memory (Kim et al., 2015). Participants performed a category learning task in which feedback was delivered immediately or after a delay, under stress and non-stress conditions. The results show that stress did not hamper probabilistic category learning in either of the feedback conditions. This pattern of results was observed even while including participants who were the least responsive to stress.

As hypothesized, the MAST manipulation elicited higher levels of stress among participants assigned to the stress condition compared to those in the non-stress condition. Specifically, there were no differences in stress levels between the different groups before the stress manipulation, whereas a significant difference was observed between them after the stress manipulation was delivered. This was reflected in both subjective (self-report questionnaires) and objective (GSR) measures. These findings are consistent with previous demonstrations of increased skin conductivity after exposure to either socially evaluated cold-pressor test manipulation (Schwabe et al., 2008b; Bhanji et al., 2016) or other types of stress manipulations, such as the Trier Social Stress Test (TSST) (Guez et al., 2016), compared to non-exposed groups. It is also consistent with studies showing an increase in self-reported stress levels due to manipulations that induce stress, such as MAST and SECPT (Schwabe et al., 2008b; Schwabe and Wolf, 2010; Smeets et al., 2012).

Probabilistic category learning was not affected by stress, in conditions that encouraged striatal-based learning (immediate feedback) or hippocampal-based learning (delayed feedback). This state of affairs was apparent in both the learning and test phases. The finding that probabilistic category learning was not impaired under stress in the immediate feedback condition is consistent with previous findings showing that stress does not impair striatal-based learning. For example, although a psychosocial stress manipulation led to the use of procedural learning strategies in the study conducted by Schwabe and Wolf (2012), participants’ performance on the feedback-based learning task was not influenced by stress. This pattern of results was also observed in the study conducted by Wirz et al. (2017), in which feedback timing was not manipulated experimentally but varied across studies due to changes in temporal resolution of the measured signals (two different methods, EEG vs. fMRI, were used to quantify brain activity during a probabilistic category learning task). Consistently, stimulus-response learning based on trial and error was not influenced by stress in the study conducted by Vogel and Schwabe (2018). Similarly, procedural reinforcement learning was found to be unaffected by stress (Petzold et al., 2010). Our findings are therefore consistent with these studies. On the other hand, the lack of influence of stress on learning in the delayed feedback conditions contradicts previous findings demonstrating impaired performance on tasks that rely on the hippocampus under stress (for a review see, Kim et al., 2015). In particular, stress was found to impair hippocampal-based spatial and contextual memory tasks (Schwabe et al., 2009). In addition, in tasks that required free recall of word lists or paragraph memorizing, performance was found to significantly deteriorate under stress (Newcomer et al., 1994; Kuhlmann et al., 2005). Note, however, that not all hippocampal memory types are negatively influenced by stress. For example, rats exposed to stress display enhanced hippocampus-dependent contextual fear conditioning (Cordero et al., 2003). In addition, stress was actually shown to enhance instructed stimulus-response learning (Vogel and Schwabe, 2018), which is considered a hallmark of goal-directed hippocampal-based learning. Vogel and Schwabe (2018) pointed out that the lack of influence of stress on this type of learning may have stemmed from the fact that the task posed no burden on working memory. Similarly, in the present study, working memory demands were minimized, as the chosen outcome and stimuli remained on screen during the delay period. It is therefore possible that stress impairs performance in tasks that rely on working memory (e.g., recalling a paragraph/list of words), a notion that is consistent with reports that stress impairs performance in working memory tasks (Kuhlmann et al., 2005). As mentioned earlier, another important factor when considering whether stress enhances or impairs hippocampal-based learning is timing. For example, the time point in which stress is delivered (acquisition, consolidation, retention) or the time interval between stress induction and learning task. If stress appears in close temporal proximity to the given learning episode, it is likely to enhance learning, whereas distant temporal proximity between stress and learning is likely to impair learning. In the current study, we examined the influence of stress on the encoding rather than the retrieval phase of memory, and there was no gap between stress induction and learning episode. Therefore, stress was delivered in close temporal proximity to the learning and test episodes. This state of affairs could explain why we did not observe a deterioration in performance under the delayed feedback conditions. Future investigations should continue examining the conditions by which stress would exert a negative influence on memory performance when feedback is delayed. It might be that delaying feedback does not influence current encoding but could impair later memory retrieval. It is also possible that increasing the time interval between stress induction and learning could change the results. Nevertheless, the current results add to previous literature by showing that performance in which a manipulation of feedback delay is presented and stress is delivered in close temporal proximity to the encoding episode, is resistant to the negative influence of stress. These findings suggest that not all types of hippocampal-based learning are negatively influenced by stress.

The literature is still unclear as to whether the contradictory effects of stress on multiple memory systems are indirect, that is, arise as the result of stress that decreases the hippocampus’ ability to interact (e.g., compete) with other memory systems, or of a direct enhancement effect on non-hippocampal memory systems (Kim et al., 2015). The present results suggest no definitive answer to this question but nonetheless are more supportive of the first possibility. In the current study, we observed no group differences when the training experience encouraged reliance on striatal learning mechanisms (when feedback was immediate), that is, learning was not enhanced in the stress group compared with the non-stress group. This pattern of results may suggest that when learning does not rely on the hippocampus, performance is not enhanced under stress but rather remains intact. This may suggest that previous observations in the literature reporting group differences between stress and non-stress groups may have arisen from a reduced ability of the hippocampus to compete with other memory systems rather than from the enhancement of non-hippocampal memories. It should be noted that although we cannot clearly dissociate procedural from declarative memory in the present task, the manipulation we used (delay of feedback) encourages greater reliance on declarative memory systems compared with procedural memory systems (Foerde and Shohamy, 2011; Arbel et al., 2017; Lighthall et al., 2018). Future studies are required to further characterize the trade-off between striatal vs. hippocampal-based memory systems in the context of stressful responses.

It might be the case that the influence of stress on memory systems as a function of feedback timing is not traceable at the behavioral level but could be manifested by using more sensitive brain-level measures. In fact, feedback timing in some cases does not influence behavioral performance among neurotypicals but modulates the engagement of memory systems at the neural level (Foerde and Shohamy, 2011). A similar pattern is observed when considering the influence of stress on procedural learning (Schwabe and Wolf, 2012). Another potential avenue for exploration is to investigate participants’ strategy use during learning under stress or to include a declarative memory test at the end of the learning episode. In the study conducted by Foerde and Shohamy (2011) a surprise memory test was included at the end of the probabilistic learning task, in which participants were required to indicate which of two outdoor photos appeared during the training in the probabilistic learning task. Inclusion of such an episodic memory test in future studies could help further clarify which types of hippocampal-based memories are negatively influenced by stress.

The present study has several limitations. First, we did not include cortisol measurements, which are highly relevant for stress-induced shifts from hippocampal to striatal learning (Schwabe and Wolf, 2013). By measuring cortisol levels one might be able to identify participants in whom cortisol levels were not elevated by the stress manipulation. In addition, we did not assess several factors that could affect the hypothalamic–pituitary–adrenal responsivity to stressors, such as smoking, estrous cycle phase in females, Body Mass Index, or medication intake, which could influence the results. In addition, although there are significant group differences with regard to the objective and subjective measures, it is still possible that the manipulation we used was not sufficient to produce a behavioral effect. We believe that this possibility is less likely, as differences in behavioral performance were previously reported in studies that used the same stress manipulation as we did (Meyer et al., 2013; Quaedflieg et al., 2013).^3^ In addition in the current study, similar to the study conducted by Foerde and Shohamy (2011), trial duration between the immediate and delayed feedback conditions differed. In some studies trial duration is equated by introducing a longer inter trial interval for the immediate feedback condition (Maddox et al., 2003). Since the intertrial interval is introduced after participants’ response and feedback presentation, it could only affect the following trial. Since we employed a mixed design, this should not differentially influence the different feedback conditions. Shorter trial duration in the previous trial might bias participants toward a faster response in the current trial and this might in turn have a differential effect on procedural vs. declarative memory systems (Smith et al., 2015). In Smith et al.’s (2015) study, participants were required to learn categories under unspeeded or speeded conditions. The results showed that speeded conditions impaired implicit but not explicit category learning. Since performance in our task was rather high in the immediate feedback condition, which is presumed to engage the procedural memory system (above 80% on average), it seems that our task did not bias participants toward a speeded response. If participants were inclined toward a speeded response, we would expect to observe lower performance in the immediate feedback condition. Finally, the present study rests on the assumption that feedback timing modulates the engagement of the different memory systems. Although animal research (Roesch et al., 2007) and human behavioral (Maddox et al., 2003; Maddox and David, 2005; Opitz et al., 2011; Chandrasekaran et al., 2014), neural (Foerde and Shohamy, 2011; Arbel et al., 2017; Lighthall et al., 2018), and patient studies (Foerde et al., 2013a, b) support such a notion, more studies are needed in order to verify this claim. Currently, imaging studies might be influenced by feedback processing and the differences in the neural activation observed in these studies might not represent differences in the engagement of the different memory systems.

It is commonly thought that hippocampal-based learning is negatively influenced by stress. In the present study we examined the influence of stress using the MAST manipulation on probabilistic category learning under different conditions that favor either striatal-based (immediate feedback) vs. hippocampal-based (delayed feedback) learning. Stress did not impair learning in either of the feedback conditions. This study suggests, therefore, that not all hippocampal-based learning types are necessarily impaired by stress.

## Data Availability Statement

The datasets generated for this study are available on request to the corresponding author.

## Ethics Statement

The studies involving human participants were reviewed and approved by the Ethics Committee of the Faculty of Education at the University of Haifa. The patients/participants provided their written informed consent to participate in this study.

## Author Contributions

YG programmed the experiments. RB collected the data. YG and RB analyzed the data and wrote the manuscript.

## Conflict of Interest

The authors declare that the research was conducted in the absence of any commercial or financial relationships that could be construed as a potential conflict of interest.

## Footnotes

1 https://jasp-stats.org

2 When entering a gender variable into the analyses, there was only a marginally significant main effect of gender indicating that males performed better than females at the test phase, *F*(1,59) = 3.89, *p* = 0.053; η*p^2^* = *0.06*; no other significant main effects or interactions with gender were observed, either in the learning phase or in the test phase (minimum *p* = 0.204).

3 The stress manipulation that we used was identical to the study of Quaedflieg et al. (2013) in terms of the way stress was induced (the MAST), the time point in which stress was delivered (encoding phase), the time point in which subjective stress ratings were obtained, as well as the interval between stress induction and the task (at least for the proximate stress group). The only difference between the studies was the task employed, since the theoretical question we wanted to address was different than that investigated in the study of Quaedflieg et al. (2013). In order to compare the results from the study conducted by Quaedflieg et al. (2013) to those of our study, we calculated the stress manipulation’s effect size (subjective ratings of stress after the stress manipulation minus ratings before the stress manipulation of the stress group) for both studies. The calculated effect size in Quaedflieg et al.’s (2013) study was calculated by using the reported mean difference and the standard error of the mean (SEM) of the proximate stress group. The effect size in their study was medium to large (Cohen’s *D* = 0.75). The effect size in our study was much larger (Cohen’s *D* = 2.04), which indicates that our participants were highly responsive to the stress manipulation, even more so than the participants in the study conducted by Quaedflieg et al. (2013).
