# Supplementary material for: Does Acute Stress Impact Declarative and Procedural Learning?
Source: Front Psychol. 2020 Mar 26;11:342. doi: 10.3389/fpsyg.2020.00342 (PMC7113394; doi:10.3389/fpsyg.2020.00342)
Supplement: Supplementary file 1 [file Data_Sheet_1.docx]

**Supplementary material**

The participants completed the following questionnaire during three different time points across the experiment (before/after the stress manipulation and at the end of the task). Before the stress manipulation they were required to answer the questions based on their current feeling. After the stress manipulation they were required to answer the questionnaire concerning how they felt during the MAST. In the last time point they were required to answer the questionnaire based on how they felt during the probabilistic category learning task. The questionnaire was developed at the stress and psychopathology lab at the University of Haifa and was provided to the authors by Dr. Roee Admon.

| 5  I was so stressed out that I could not think | 4  I was very stressed | 3  I was moderately stressed | 2  I was a little stressed | 1  I was not stressed at all |  |  |
| --- | --- | --- | --- | --- | --- | --- |
|  |  |  |  |  | To what extent did you feel stressed during the task? | 1 |
| Very much | Largely | Moderately | Slightly | Not at all |  |  |
|  |  |  |  |  | To what extent do you think that stress impaired your performance on the task? | 2 |
|  |  |  |  |  | To what extent did you feel that you were tense during the task? | 3 |
|  |  |  |  |  | To what extent did you feel aroused during the task? | 4 |
|  |  |  |  |  | To what extent did you experience discomfort during the task? | 5 |
|  |  |  |  |  | To what extent did you feel a sense of control during the task? | 6 |
|  |  |  |  |  | To what extent did you feel that the task was difficult for you? | 7 |
|  |  |  |  |  | To what extent did you suffer during the task? | 8 |
